# Winner of the Ronald Melzack–Canadian Journal of Pain Paper of the Year Award for 2024/Récipiendaire du Prix Ronald Melzack Pour L’Année 2024 Des Articles Parus Dans La Revue Canadienne de La Douleur

**DOI:** 10.1080/24740527.2025.2544489

**Published:** 2025-09-04

**Authors:** Joel Katz, Heather Lumsden-Ruegg, Anna Waisman

**Affiliations:** Editor-in-Chief, Canadian Journal of Pain; Editorial Assistant, Canadian Journal of Pain; Editorial Manager, Canadian Journal of Pain

The *Canadian Journal of Pain* and the Canadian Pain Society confer an award, annually, in memory of the late Ronald Melzack, a Canadian psychologist known for his trailblazing theoretical and empirical contributions to the field of pain research and management. This year, the Ronald Melzack–*Canadian Journal of Pain* Paper of the Year Award for articles published in 2024 was presented to Cynthia J. Thomson, Hanna Pahl, and Luisa V. Giles from the University of the Fraser Valley for their article titled “Randomized Controlled Trial Investigating the Effectiveness of a Multimodal Mobile Application for the Treatment of Chronic Pain.”^1^ The winning paper was selected by the Editor-in-Chief from a short list of five original articles published in the *Journal* in 2024 based on rankings by 24 *Journal* Editorial Board members who considered originality, novelty, quality, and potential impact in their submissions. The Thomson et al. article was the clear winner based on the number of Editorial Board members who ranked it as their top choice. The Paper of the Year Award for 2024 was presented by Joel Katz to Dr. Thomson, who accepted it on behalf of her coauthors at the 45th Annual Scientific Meeting of the Canadian Pain Society in Toronto on May 3, 2025.

Ideal care for people with chronic pain is evidence-based and multidisciplinary, addressing the biological, psychological, and social aspects of life that are known to influence the experience of pain. Despite what is known about the importance of providing care to people living with chronic pain, individuals face notable challenges gaining access to the multidisciplinary care they need.^2^ These challenges include a “treatment gap” driven by issues of scalability—ensuring that treatments are available to large numbers of individuals in need—and reach—providing care to individuals who have traditionally lacked access.^3^ Comprehensive, multidisciplinary pain treatment programs are expensive to implement and difficult to scale, contributing to systemic barriers (e.g., long wait times) for patients who might otherwise benefit. The widespread uptake of smartphone technology has made digital options in pain treatment possible, and digital pain apps are now being used to monitor pain and pain-related outcomes, improve symptom self-management, and connect patients with health care providers. Though such tools show promise in addressing some of the care gaps experienced by individuals living with chronic pain, not all digital pain apps address the biopsychosocial needs of their users. A systematic review conducted by MacPherson and colleagues^4^ identified more than 500 digital pain apps available in the Canadian market, yet only 12 included content pertaining to the psychological aspects of pain and only one had undergone independent scientific testing for efficacy. High-quality digital interventions could play a vital role in addressing the unmet needs of people living with chronic pain, yet there is a paucity of research evidence for the efficacy and effectiveness of most apps available for download. In light of the rapidly growing prominence of eHealth in the current healthcare landscape, the *Journal* has recently launched a call for manuscripts featuring an article collection on digital health and pain management.

The winner of the Ron Melzack Paper of the Year Award for 2024 is a timely contribution to the literature documenting the use of digital pain apps. Thomson and colleagues^1^ conducted a randomized controlled trial to assess the efficacy of 6 weeks of mobile app engagement for the treatment of chronic pain. Participants were 198 adults reporting nonmalignant chronic pain lasting at least 6 months, randomized to an intervention group using the Curable app or a waitlist control, usual care group with delayed app access at the end of the study. Participants in the intervention group were asked to engage with the app a minimum of four times per week in one activity of their choosing, with options including audio lessons in neuroscience pain education, cognitive-behavioral therapy–based lessons, meditation, expressive writing, and podcast listening. Surveys were completed before and after 6 weeks of app use or usual care, assessing pain severity (and intensity) and pain interference as primary outcomes, along with medication use and various pain-related factors (e.g., pain catastrophizing, anxiety, quality of life) as secondary outcomes. Results were analyzed by a linear mixed model evaluating Group, Time, and Group × Time interaction effects. The Group × Time interaction effect was significant for pain severity, pain interference, pain catastrophizing, depression, anxiety, and stress scores, pointing to greater improvements in the intervention group compared to the usual care group after 6 weeks. Significant improvements in the intervention group relative to baseline were evident at 12 weeks. The authors concluded that engaging with the app is an affordable pain management option and a scalable alternative to in-person programs, which often have long waitlists. Noted limitations of the study include its remote delivery, reliance on self-reported data, and the relatively short duration of the intervention.

On behalf of the *Journal* Editorial Board and the Canadian Pain Society Awards Committee, we offer our most heartfelt congratulations and appreciation to Dr. Thomson and colleagues for their winning article and for submitting their manuscript to the *Canadian Journal of Pain*. We look forward to next year’s Annual Meeting of the Canadian Pain Society when we hope to announce two Paper of the Year Awards: one for the applied/clinical sciences and the other for the fundamental/preclinical sciences. Please submit your original research to the *Canadian Journal of Pain* and perhaps you’ll be reading about your award-winning article in these pages a year from now!
